# Extracellular Neural Microstimulation May Activate Much Larger Regions than Expected by Simulations: A Combined Experimental and Modeling Study

**DOI:** 10.1371/journal.pone.0041324

**Published:** 2012-08-07

**Authors:** Sébastien Joucla, Pascal Branchereau, Daniel Cattaert, Blaise Yvert

**Affiliations:** 1 Université Bordeaux, Institut des Neurosciences Cognitives et Intégratives d'Aquitaine, UMR5287, Bordeaux, Talence, France; 2 CNRS, Institut des Neurosciences Cognitives et Intégratives d'Aquitaine, UMR5287, Bordeaux, Talence, France; University of Sheffield, United Kingdom

## Abstract

Electrical stimulation of the central nervous system has been widely used for decades for either fundamental research purposes or clinical treatment applications. Yet, very little is known regarding the spatial extent of an electrical stimulation. If pioneering experimental studies reported that activation threshold currents (TCs) increase with the square of the neuron-to-electrode distance over a few hundreds of microns, there is no evidence that this quadratic law remains valid for larger distances. Moreover, nowadays, numerical simulation approaches have supplanted experimental studies for estimating TCs. However, model predictions have not yet been validated directly with experiments within a common paradigm. Here, we present a direct comparison between experimental determination and modeling prediction of TCs up to distances of several millimeters. First, we combined patch-clamp recording and microelectrode array stimulation in whole embryonic mouse spinal cords to determine TCs. Experimental thresholds did not follow a quadratic law beyond 1 millimeter, but rather tended to remain constant for distances larger than 1 millimeter. We next built a combined finite element – compartment model of the same experimental paradigm to predict TCs. While theoretical TCs closely matched experimental TCs for distances <250 microns, they were highly overestimated for larger distances. This discrepancy remained even after modifications of the finite element model of the potential field, taking into account anisotropic, heterogeneous or dielectric properties of the tissue. In conclusion, these results show that quadratic evolution of TCs does not always hold for large distances between the electrode and the neuron and that classical models may underestimate volumes of tissue activated by electrical stimulation.

## Introduction

Extracellular electrical stimulation of neural tissues has been used for decades, for either fundamental research purposes or clinical treatment applications. Macroscopic stimulation using millimeter-scale electrodes is used in a number of clinical applications, including suppression of tremors in Parkinson's disease using deep brain stimulation (DBS) [1], restoration of auditory perception using cochlear implants [2], [3], and alleviation of chronic pain [4] or restoration of motor functions [5], [6] using spinal cord stimulation. Over the past decade, smaller electrodes with typical size of the order or below 100 microns have been assembled into microelectrode arrays (MEAs). These multichannel probes allow interfacing large neural networks with hundreds of recording and stimulating sites. These new devices have triggered a surge towards finding pertinent paradigms of extracellular microstimulation to modify and even control the dynamics and plasticity of neural networks [7], [8], [9], and also, in clinical applications, to restore visual perception using retinal implants [10], [11] or to create bidirectional brain-machine interfaces [12], [13], [14]. For all these applications, a key step is to control the spatial extent of an electrical stimulation, which often remains an open question.

Two types of studies have been carried out previously to estimate the spread of activation of an electrical stimulation: either experimentally or using simulations. Pioneering experimental studies carried out several decades ago have suggested that the threshold current required to elicit an action potential in a cell or a fiber is proportional to the square of the distance to the electrode [15], [16], [17], [18], [19], [20]. This current-distance relationship has been verified for electrode-neuron/fiber distances smaller than a few hundreds of microns, and there is no experimental evidence that this quadratic law remains valid for larger distances. Moreover, in these studies, the exact position of the electrode with respect to the full arborization of the cell is often difficult to determine for obvious experimental constraints. More recently, modeling studies have been used to numerically estimate the neural response to an electrical stimulation. The particular advantage of these approaches is to offer a very flexible way to predict the volumes of activated tissue (VAT) for various electrode configurations, neuronal morphologies and conductive media. It has been shown that VATs estimated with simulation approaches fit with previous experimental recordings of the literature for distances below about 200–300 microns [21], [22]. Indirect validation of simulation results have also been reported by others, by correlating modeling predictions with clinical data [23]. Based on these results, simulation approaches are expected to accurately predict VATs over large distances of several millimeters [24]. However, to our knowledge, there has been no direct comparison, within the same study, between experimental and modeling prediction of the spatial extent of extracellular electrical stimulation. Moreover, the validation of computational approaches over distances of several millimeters has not been performed. This is of primary importance for instance in light of DBS paradigms aiming at stimulating millimeter-scale regions of the central nervous system (CNS) using currents of the order of the mA [25].

The purpose of this study was thus to confront, in a common paradigm, experimental and modeling determination of the spread of extracellular neural stimulation over distances encompassing several millimeters. First, we determined experimentally the direct activation thresholds of patch-clamp-recorded spinal motoneurons subject to electrical microstimulations using MEAs. Distances up to 3 millimeters were considered. Second, we built a model mixing finite elements and compartmentalized neurons (as introduced in a previous study [26]), corresponding to the experimental paradigm. We found that, while experimental and simulation thresholds closely match for short electrode-neuron distances, computational models strongly overestimate (by two orders of magnitude) these thresholds at large distances.

## Materials and Methods

### 1. Experimental protocols

Using whole-cell patch-clamp recordings of spinal cord motoneurons and microelectrode arrays dedicated to *in vitro* experiments, we characterized the spatial extent of current-controlled extracellular electrical microstimulations. All experimental protocols conformed to recommendations of the European Community Council Directive of November 24, 1986 (86/609/EEC) and local French legislation for care and use of laboratory animals.

#### a. Microelectrode array (MEA)

**Figure 1:**
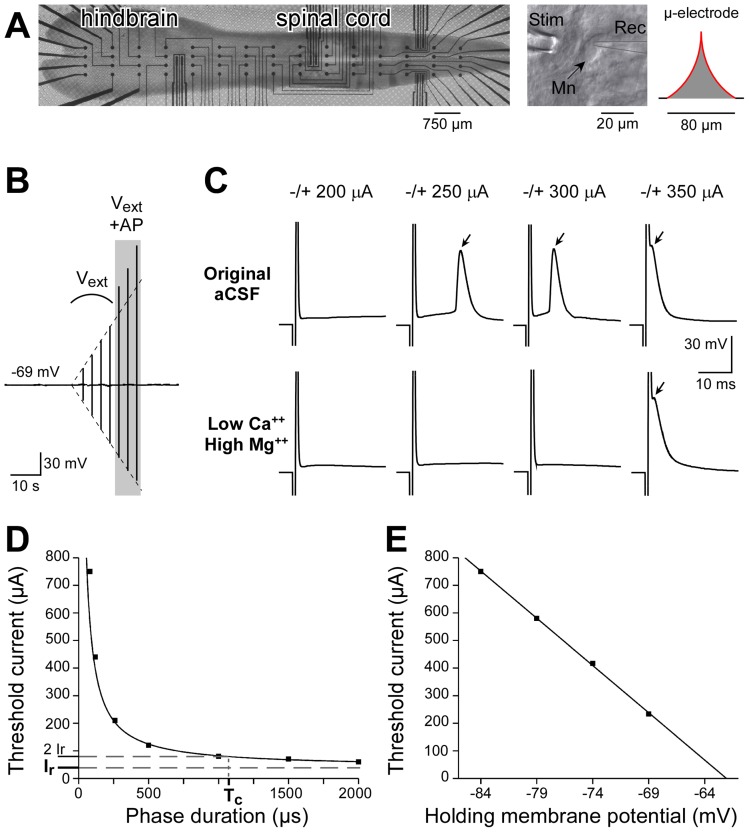
Experimental protocol of extracellular stimulations. **A**: Dorsally-opened hindbrain-spinal cords of (*n* = 9) E14.5 mouse embryos were positioned on MEAs, with the side of the central canal facing the 4×15 microelectrodes of the array (left). In each preparation, one motoneuron was patch-clamped in whole cell configuration, and stimulated using both a glass pipette positioned close to its cell body (middle) and the microelectrodes of the MEA. The theoretical shape of the 3D microelectrodes (height: 80 µm, base diameter: 80 µm) is shown on the right (the conductive part is in red, the insulated part is in black). **B**: Current-clamp recording during a series of stimuli with increasing intensities. In order to perform all trials in the same conditions, a slow holding current (time constant: 5 s) was injected to maintain *V_m_* at a corrected value of about −69 mV. The dashed line highlights the fact that the amplitude of the signal increases linearly with the pulse intensity (here steps of 50 µA) until an action potential is elicited which superimposes on top of the extracellular potential (3 shaded events). **C**: Threshold currents (TCs) were determined by increasing regularly the intensity of the cathodic-first biphasic current-controlled pulse. Under standard aCSF, indirect activations of the neuron were observed (top row), while only direct activations were achieved using a low Ca^2+^/high Mg^2+^ aCSF solution (bottom row). **D**: The motoneuron chronaxie (*T_c_*) was calculated by determining TCs for different phase durations and fitting the hyperbolic Weiss's law [32]. On average, we found *T_c_* = 913±132 µA (*n* = 3). Based on this value, we chose a phase duration of 1 ms for all experiments. **E**: Activation thresholds decreased linearly with holding membrane potential values.

Extracellular electrical stimulations were delivered to whole hindbrain-spinal cord preparations of mouse embryos using MEAs, which were composed of a 8 2D electrodes and a grid of 4×15 3D microelectrodes (width spacing: 250 µm, length spacing: 750 µm, see Figure 1A, left), all made of Pt (*Ayanda Biosystems – now Qwane Biosciences –,* Lausanne, Switzerland). The 2D microelectrodes had a rectangular shape (60 µm×250 µm), and their impedance, measured at 1 kHz in a saline solution, was in the range 60–100 kΩ. The 3D microelectrodes, obtained by standard isotropic photolithography [27], had a height of about 80 µm and a base diameter of about 80 µm, all the electrode being in electrical contact with the tissue (see red line in Figure 1A, right). According to this shape, these 3D microelectrodes had a theoretical surface of 7285 µm^2^. Their impedance was in the range 100–150 kOhm. The array was surrounded by a home-made Sylgard square chamber (side length: 2 cm, height: 3 mm), and the bottom part, including electrode leads, was insulated from the extracellular medium by a 5-µm-thick SU-8 epoxy layer [27] (black line in Figure 1A, right). An external cylindrical Ag/AgCl ground electrode pellet (diameter: 2 mm, height: 4.3 mm, *World Precision Instruments*, Aston, England) was used for the return of the stimulation current.

#### b. Tissue preparation

Hindbrain-spinal cord preparations were dissected as described previously [26], [28], [29], [30]. E14.5 embryos were surgically removed from pregnant OF1 mice (*Charles River Laboratories*, L'Arbresle, France) previously killed by cervical dislocation. The whole spinal cord and medulla were dissected in a cool (6–8°C) artificial CSF (aCSF) solution (pH 7.5) composed of (in mM): 113 NaCl, 4.5 KCl, 2 CaCl_2_2H_2_O, 1 MgCl_2_6H_2_O, 25 NaHCO_3_, 1 NaH_2_PO_4_H_2_O and 11 D-Glucose) gassed with carbogen (95% O_2_ and 5% CO_2_). The spinal cord and hindbrain were opened dorsally (open-book preparation) and meninges were removed. This preparation was then placed on the MEA (Figure 1A left), with the side of the central canal facing the microelectrodes of the array, and stabilized by a plastic net with small holes (70×70 µm^2^) in order to achieve a tight and uniform contact with the microelectrodes. The neural tissue was continuously perfused at a rate of 1.5 ml/min. Experiments were performed at room temperature (22±3°C).

#### c. Whole-cell current-clamp recordings

Spinal motoneurons (*n* = 9) were identified as previously reported [31], according to their morphological features (pear-shaped large cell body) and their disposition in columns in the lumbar ventral horn. An Olympus BX51WI microscope (*Micro Mécanique*, Évry, France) equipped with differential interference contrast (DIC) and a CCD camera (SPOT RT-SE6, *Diagnostic Instruments*, Sterling Heights, MI, USA) were used to visualize motoneurons. Patch-clamp electrodes were constructed from thin-walled single-filamented borosilicate glass (outer diameter: 1.5 mm; *Harvard Apparatus*, Les Ulis, France) using a two-stage vertical microelectrode puller (PP-830; *Narishige*, Tokyo, Japan; first step: 60.1, second step: 49.6). Their resistances ranged from 4 to 6 MΩ. Electrodes were filled with Neurobiotin (0.4%) (*CliniSciences*, Montrouge, France) diluted in the following medium (mM): 5 NaCl, 130 K-gluconate, 1 CaCl_2_2H_2_O, 10 Hepes, 2 ATP Mg^2+^, 10 EGTA (pH adjusted to 7.4 with KOH). Patch-clamp electrodes were positioned on visually identified motoneurons (Figure 1A middle) using motorized micromanipulators (*Luigs & Neumann*, Ratingen, Germany). Whole-cell recordings were performed after a high-resistance seal was achieved (between 10 and 16 GΩ). Recordings were made using an Axon Multiclamp 700B amplifier (*Molecular Devices*, Sunnyvale, CA, USA), with reference to the external cylindrical ground electrode. Data were low-pass filtered (3 kHz), sampled at 50 kHz (Micro 1401, *Cambridge Electronic Design (CED)*, Cambridge, UK) and acquired with the Spike2 software (v5.14, CED). Measurements of the somatic membrane potential (*V_m_*) were corrected for liquid junction potentials (−14 mV). In order to perform all trials in the same conditions, a slow holding current (time constant: 5 s) was injected to maintain *V_m_* at a corrected value of about –69 mV (maximal variations recorded at rest: ±2 mV). This value was chosen so as to maintain *V_m_* close enough to the neuron spike threshold (about 10 mV below threshold) in order to be able to activate the neuron at low currents, but far enough to avoid spontaneous firing. We verified that activation thresholds decreased almost linearly for increasing holding membrane potentials (Figure 1E).

#### d. Direct neuronal activation with extracellular electrical stimulations

After establishment of the whole-cell configuration, the original aCSF was replaced by a low calcium/high magnesium solution (composed of (in mM): 111.85 NaCl, 4.5 KCl, 0.1 CaCl_2_2H_2_O, 5 MgCl_2_6H_2_O, 25 NaHCO_3_, 1 NaH_2_PO_4_H_2_O and 11 D-Glucose), in order to block synaptic transmission and avoid network-induced activation (see Figure 1C). Current-controlled monopolar stimulations were applied between the 3D or 2D microelectrodes of the array and the external ground electrode. A glass pipette with a tip diameter of about 10-20 µm was also used to deliver electrical stimulations as close as possible to the recorded motoneuron cell body (32±3 µm, *n* = 5, see Figure 1A middle). Stimuli were delivered using the STG2008 stimulator controlled by the MC_Stimulus II v2.1.4 software (*Multi Channel Systems*, Reutlingen, Germany). We used trains of cathodic-first biphasic current pulses separated by 3 sec (phase duration: 1 ms, chosen accordingly to motoneuron chronaxie, see below). For each stimulation site, activation thresholds were determined by delivering stimulations of increasing intensities, from 50 µA to 800 µA (maximal value allowed by the stimulator) in steps of 50 µA (see Figure 1B). The sequence of stimuli was stopped when three consecutive action potentials were triggered (see shaded area in Figure 1B), and the activation threshold current (TC) was defined as the current for which the neuron triggered the first action potential. If the TC was less than 200 µA, a more precise threshold value was determined with steps of 10 µA. Stimulation sites of the MEA were systematically explored, from the closest to the farthest. At the end of the stimulation procedure (which generally lasted between one and two hours), we verified that the neuron excitability had not changed by determining again a TC with a stimulation site close to the neuron.

#### e. Motoneuron chronaxie

In order to determine the appropriate phase duration of the pulses, extracellular stimulations of variable durations were initially delivered to 3 motoneurons. TCs were determined for different durations (60, 120, 250, 500, 1000, 1500 and 2000 µs), and the threshold-duration curve was fitted according to the hyperbolic Weiss's law [32]:


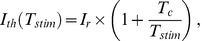
(1)

where *T_stim_* is the stimulation duration, *I_th_* is the current activation threshold, *I_r_* is the neuron rheobase and *T_c_* is the neuron chronaxie. This is further illustrated in Figure 1D, with a neuron for which we found a chronaxie of 1037 µs. On average, we determined a chronaxie of 913±132 µA (mean ± s.e.m., *n* = 3). Based on this value, a 1-ms phase duration was chosen for all experiments.

#### f. Activation threshold analysis

Our main goal was to determine the evolution of TCs with distance from the stimulation electrode. The signal recorded by the patch-clamp pipette is the sum of two components: the extracellular potential *V_ext_* due to the stimulation and the motoneuron membrane potential (see Figure 1B). During the 2-ms-long stimulus, the signal mainly reflected the extracellular electrical potential (namely, the stimulation artifact), which was characterized by a return to baseline with a short time constant, of the order of hundreds of µs (see for instance Figure 1C, *I* =  −/+ 200 µA). After the end of the stimulus, action potentials elicited by suprathreshold stimulations were undoubtedly detected, even when action potential peaks occurred before the end of the stimulus (see arrows in Figure 1C). Furthermore, the value of *V_ext_* corresponding to the TC was also measured at the end of the cathodic phase of the stimulus. The distance between the motoneuron cell body and each stimulation electrode was calculated using 2D images taken in the focal plane using the CCD camera and the ×10 microscope objective. The 3D distance between the cell body and the tip of the electrode was then calculated from its 2D position and altitude above the MEA substrate. The TCs and threshold *V_ext_* values determined for 9 motoneurons were analyzed together, data being binned into distance classes. In each class, averages and standard errors were determined.

#### g. Immunolabeling

After each experiment, immunostaining was processed on the whole spinal cord in order to reveal the morphology of the recorded E14.5 neuron (*n* = 9/9) as well as the location of voltage-gated sodium channels (*n* = 3/9). The spinal cord was fixed in 2% paraformaldehyde for 1 h at 4°C, and then rinsed three times with 0.1 M PBS (composed of (in mM): 77 Na2HPO4, 23 NaHPO, 154 NaCl, pH: 7.4). It was then immerged in 0.1 M PBS containing 10% goat serum and 0.4 Triton X-100 (T 8787, *Sigma*, St Louis, MO, USA), and incubated for 12–24 h at 4°C with a mouse monoclonal anti-sodium channel antibody (Pan Nav antibody, S 8809, Sigma) and Cy3-Streptavidin (1∶400, *Invitrogen SARL*, Cergy Pontoise, France) to reveal the Neurobiotin-injected motoneuron. After three rinses (at least 30 minutes each), the spinal cord was incubated for 1 h at room temperature in a solution containing the FITC-coupled secondary antibody (Goat anti-mouse, Alexa Fluor 1∶400, *Molecular Probes*) and Cy3-Streptavidin. After three rinses in 0.1 M PBS (at least 10 minutes each) and a last rinse in distilled water, the spinal cord was mounted in a mixture containing 90% glycerol and 10% PBS to which 2.5% 1,4-diazabicyclo[2,2,2]octane (DABCO, *Sigma*) was added, in order to reduce the rate of fluorescence quenching. All incubations and rinses were performed sheltered from light.

#### h. Confocal microscopy

Preparations were imaged with a BX51 Olympus Fluoview 500 confocal microscope equipped with blue argon (488 nm) and green helium–neon (546 nm) laser sources to visualize the pan/FITC and Neurobiotin/Cy3 labelings, respectively. Neuron morphologies and sodium channels immunolabelings were acquired with a ×60 oil-immersion objective (serial optical section thickness: 0.2 µm). Images were averaged over 2 scannings (Kalman filter).

#### i. Neuron morphology reconstruction

**Figure 2:**
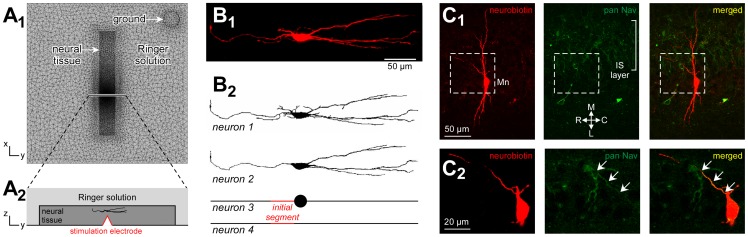
Modeling approach. Simulations were performed to compare theoretical and experimental estimations of the spatial extent of electrical microstimulation. **A**: A 3D Finite Element Model (FEM) was used to compute the electrical potential field generated in the neural tissue by an electrical stimulation. The model geometry corresponded to the experimental MEA, including the chamber, the neural tissue, one 3D electrode (which shape was approximated by a cone with a height of 80 µm and a base diameter of 80 µm) and the cylindrical ground electrode (height  = 2.7 mm, diameter  = 2 mm). This volume was subdivided into two regions, representing the aCSF solution (side length: 2 cm, height: 3 mm, conductivity *σ* = 1.65 S/m) and the neural tissue (length: 13 mm, width: 2 mm, height: 200 µm, *σ* = 0.10 S/m). **B**: The FEM-calculated extracellular potential field was then applied to compartmentalized motoneuron models in order to determine theoretical activation thresholds. Four different neurons were considered (B2), the most complex of them (1895 compartments) being a 3D reconstruction of a Neurobiotin-injected motoneuron considered in the experimental study (B1). These neuron models were equipped with passive properties in the soma, dendrites and most of the axon. Voltage-dependent Na^+^ and K^+^ channels were located in the initial segment of the axon (length = 34 µm), leaving from the soma, in accordance with the immunolabelings (C). **C**: Confocal projection of a neurobiotin-injected motoneuron (red) and anti-sodium channel labeling (pan Nav, green). C1: Global view of the neuron and the layer of initial segments (IS), oriented in the medio-lateral direction (60 optical sections). C2: Close-up in the perisomatic region (6 optical sections), revealing the localization of the sodium channels in the IS of the axon (merged image, yellow, see arrows). In *n* = 3 experiments, the IS had a length of 34 +/− 3 µm. [C: caudal; M: median; L: lateral; R: rostral].

One motoneuron was fully reconstructed to build a compartmental neural model (Figure 2B_1_, see modeling part of this study described below). For this purpose, 2 overlapping stacks of 246 confocal images were acquired at ×60 magnification (serial optical section thickness: 0.2 µm), each stack covering more than half of the whole neuron morphology. These stacks were then merged into a single stack (using the *vias* software, v2.1, freely downloadable at http://www.mssm.edu/cnic/tools-vias.html), which was then loaded into the Neurolucida software (v 6.02.2, *MBF Bioscience*, Williston, USA) to interactively reconstruct the neuron arborization. Finally, the morphology was converted to a.hoc file compatible with the NEURON software, v7.2 [33] using the cvapp software (v1.2, freely downloadable at http://www.compneuro.org/CDROM/docs/cvapp.html).

### 2. Modeling approach: General approach

We further performed simulations to compare theoretical and experimental estimations of the spatial extent of microstimulation. For that purpose, we used a classical 2-stage model made of a finite element model (FEM) for the calculation of the electrical potential field in the tissue, and a compartmentalized neuron model for the computation of the response of a neuron stimulated by this field (see [34] for a review of the biophysical and mathematical background of these models). The calculation of the potential field was performed either in the case of an homogeneous, isotropic and purely resistive tissue, or in the case of an anisotropic, heterogeneous, or leaky dielectric tissue.

### 3. Calculation of the potential field in the case of an homogeneous, isotropic and purely resistive tissue

The electrical potential field generated in the neural tissue by an electrical stimulation was computed using a 3D FEM using COMSOL Multiphysics 3.4 (COMSOL AB, Stockholm, Sweden) interfaced with Matlab 7.7 (The Mathworks, Natick, USA), under Linux (Fedora 14).

#### a. Model geometry

The 3D model geometry corresponded to the experimental MEA, including the chamber, the neural tissue, one 3D stimulation electrode and the ground electrode. The outer limits of the model corresponded to the inner geometry of the MEA square chamber (side length: 2 cm, height: 3 mm). This volume was subdivided into two regions, representing the aCSF solution and the neural tissue. The neural tissue had a parallelepipedical shape whose dimensions fitted the E14.5 embryonic mouse hindbrain-spinal cord preparation (length: 13 mm, width: 2 mm, height: 200 µm, see Figure 2A_1_). We verified that modeling a more realistic shape did not alter the distribution of the electrical potential within the tissue. A single stimulation electrode was modeled on the bottom surface of the chamber, represented by a 3D conical boundary, whose dimensions corresponded to the actual MEA used experimentally (base diameter: 80 µm, height: 80 µm, see Figure 2A_2_). The external ground electrode was modeled by a cavity inside the domain representing its immersed part in contact with the aCSF solution (diameter: 2 mm, height: 2.7 mm).

#### b. Volume equation

The finite element model solved the homogeneous Poisson equation:


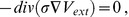
(2)

where *V_ext_* is the extracellular electrical potential. The electrical conductivities σ of the aCSF and neural tissue were supposed homogeneous and isotropic in each region. The conductivity of the aCSF was set to *σ_aCSF_* = 1.65 S/m, a value measured with a conductimeter at room temperature. The conductivity of the spinal cord was set to *σ_tissue_* = 0.10 S/m, a value close to that estimated in a previous study [26].

#### c. Boundary conditions

Insulating boundary conditions (BCs) were assigned to the circumference of the chamber, the air-aCSF solution interface (top part of the chamber), and the floor of the chamber:


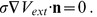
(3)

The conductive elements (ground and stimulation electrodes) were assigned Robin BCs (derived from Ohm's law at the electrode-electrolyte interface), as previously validated by comparing experimental measurements and modeling calculations of the potential field [26]:


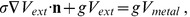
(4)

where *g* is the surface conductance of the electrode-electrolyte interface. The metal voltage in Equation 4 was set to *V_metal_* = 0 for the ground and *V_metal_* =  *V_stim_* for the stimulation electrode, with *V_stim_* adjusted to have a normalized current of 1 µA. The surface conductances of the electrodes were set respectively to *g_stim_* = 338 S/m^2^ and *g_ground_* = 975 S/m^2^ [26].

#### d. Mesh and solver

The 3D geometry of the model was densely meshed with 450628 tetrahedral Lagrange P2 elements and 629034 degrees of freedom (Figure 2A_1_), in order to minimize the numerical error on the electrical potential *V_ext_* and its derivatives. The problem was solved with the SSOR-preconditioned conjugated gradient algorithm.

### 4. Calculation of the potential field in the case of an anisotropic, heterogeneous, or leaky dielectric tissue

The influence of different electrical properties of the neural tissue on the potential field were further evaluated separately by modeling the spinal cord either with a globally anisotropic conductivity, or with local variations of the (isotropic) conductivity, or by taking into account dielectric properties of the tissue. Each of these models was extended from the original model described above, as follows:

#### a. Anisotropy

The originally isotropic conductivity of the tissue was replaced by an anisotropic model, made of a longitudinal value (in the *x* direction) equal to 0.33 S/m and a transverse value (in the *y* and *z* directions) equal to 0.083 S/m, according to a former model of the spinal cord white matter [22].

#### b. Heterogeneity

In the original model geometry, we added two series of ten 20-µm-side cubes, with an inter-cube space of 20 microns, located respectively at 500 microns and 3 millimeters from the stimulation electrode. These small domains were assigned conductivities equal to 0.1 and 10 times the original tissue conductivity (0.1 S/m), in alternation. In this model, the neuron model was positioned on the edge of these heterogeneities in order to maximize their effect on the neural response.

#### c. Dielectric effects

Dielectric properties, neglected in the quasi-static approximation leading to the Poisson equation (Eq. 2), were finally included in the neural tissue domain, which led to the resolution of the Helmholtz equation:


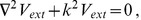
(5)

where 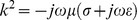 is the propagation constant, 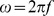 is the angular frequency of the stimulation, *μ* is the magnetic permeability of the tissue and *ε* its dielectric permittivity [34], [35]. The spectrum of the cathodic-anodic pulse used in this study displays a main component at *f*_1_ = 500 Hz and secondary harmonics at *f*_3_ = 1500 Hz, *f*_5_ = 2500 Hz, and every *f*_2*p*+1_. Their amplitude decreased in a cardinal sine so that 99% of the energy of the pulse is contained in the harmonics below 10500 Hz (81% being already contained in the main frequency 500 Hz). For that reason, we focused on the field generated at 500 Hz and 10500 Hz. The magnetic permeability of the spinal cord was set to that of the vacuum (μ = 4π×10^−7^ H/m) and its dielectric permittivity was set to ε = ε_0_×ε_r_, with ε_0_ = 8.854×10^−12^ F/m (dielectric permittivity of the vacuum) and ε_r_ = 3×10^5^ at 500 Hz and 2×10^4^ at 10500 Hz [35]. In a subsequent model, we also modeled an heterogeneity of the dielectric permittivity by adding an 80-µm-sided cube at 500 microns or 3 millimeters from the stimulation electrode, in which ε_r_ was set to 10 or 100 times its original value. In this model, the neuron was positioned on the edge of this heterogeneity in order to maximize its effect on the neural response.

### 5. Computation of the neuronal response

We used a cable equation framework to compute the membrane response of a non-myelinated compartmentalized neuron (indeed, motoneurons are not myelinated at embryonic stages) embedded in the extracellular potential field calculated as above. Simulations were run using the NEURON software, v7.2 [33].

#### a. Neuron morphologies

Four neuron morphologies of decreasing dendritic complexities were used for the compartment model (Figure 2B_2_). The most complex morphology (neuron 1, *N* = 1895 compartments) was obtained from a full reconstruction of stacks of confocal images (Figure 2B_1_), as detailed in the Experimental Methods. Neuron 2 was an approximation of neuron 1, with only its main morphological features (soma, axon and the main dendrites, *N* = 1250 compartments). Neuron 3 was a straight neuron displaying the same basic morphological characteristics as Neuron 2 (soma diameter, axon and dendrite length, 300 1-µm-long compartments). Finally, Neuron 4 was a straight fiber of same length as Neuron 3 (300 1-µm-long compartments).

#### b. Electrical characteristics

Each compartment was assigned identical passive characteristics: the surface capacitance was set to its classical value (*c_m_* = 1 µF/cm^2^), the intracellular resistivity was set to *ρ_i_* = 100 Ω.cm, which was within the range of values used in the literature (33–300 Ω.cm) [36], [37], [38], [39], [40]. Using these parameters, the surface leakage conductance was then estimated to *g_l_* = 3.10^–5^ S/cm^2^ by comparing the experimental and modeling responses of a neuron to a hyperpolarizing pulse of –50 pA. Active Hodgkin-Huxley-like [41] sodium and potassium conductances were set to the initial segment of the axon (*g_Na_* = 0.24 S/cm^2^, *g_K_* = 0.036 S/cm^2^). This initial segment, which corresponded to a 34-µm-long portion of the axon starting from the soma, was chosen in accordance with the experimental anti-sodium channel immunolabelings (see Figure 2C). In this active region, the leakage potential was calculated so that the passive leakage current compensated the non-zero sodium and potassium active currents at rest, hence imposing a uniform value of the resting potential over the neuron (*E_l_* =  +13.88 mV, *E_Na_* =  +50 mV, *E_K_* =  –77 mV, *V_rest_* =  –69 mV).

#### c. Extracellular stimulations

The extracellular potential field *V* computed with the FEM for a nominal current of *I* =  +1 µA was interpolated at the center of each compartment *j* (*V_j_*) and assigned with the extracellular mechanism in NEURON. As in the experimental protocol, cathodic-first biphasic stimulations with phase duration of 1 ms were used, that is the extracellular potential of compartment *j* was 0 before the stimulus, –*I×V_j_* during the cathodic phase, +*I×V_j_* during the anodic phase, and 0 after. Starting from *I* = 1, the amplitude *I* of the stimulus was recursively doubled until firing an action potential in the soma (detected after the end of the anodic phase, as follows: An action potential occurred when the membrane potential *V_m_* at time *t_k_* was greater than *V_m_* at times *t_k−1_* and *t_k+1_*, and above a given threshold, set to –20 mV). Then, the current *I* was successively decreased and increased by dichotomy until finding the threshold current, with a relative precision of 0.1%. A 50-µs time step was used, allowing a reduced error on the activation threshold estimation (using a 10 times smaller time step led to a threshold difference of less than 1%).

#### d. Threshold-distance curves

In these simulations, the neurons were oriented parallel to the *y*-axis in order to mimic the position of experimentally recorded motoneurons, which laid perpendicular to the central canal of the spinal cord (confocal observations). They were moved along the *x*-axis (in the direction of the spinal cord) at *z* = 130 µm, in order to determine the evolution of the activation thresholds as a function of the electrode-neuron distance. The height of 130 µm (about 50 µm above the electrode tip) corresponded to the actual depth of the experimentally stimulated motoneurons.

## Results

### 1. Experimental determination of threshold current-distance curves

**Figure 3:**
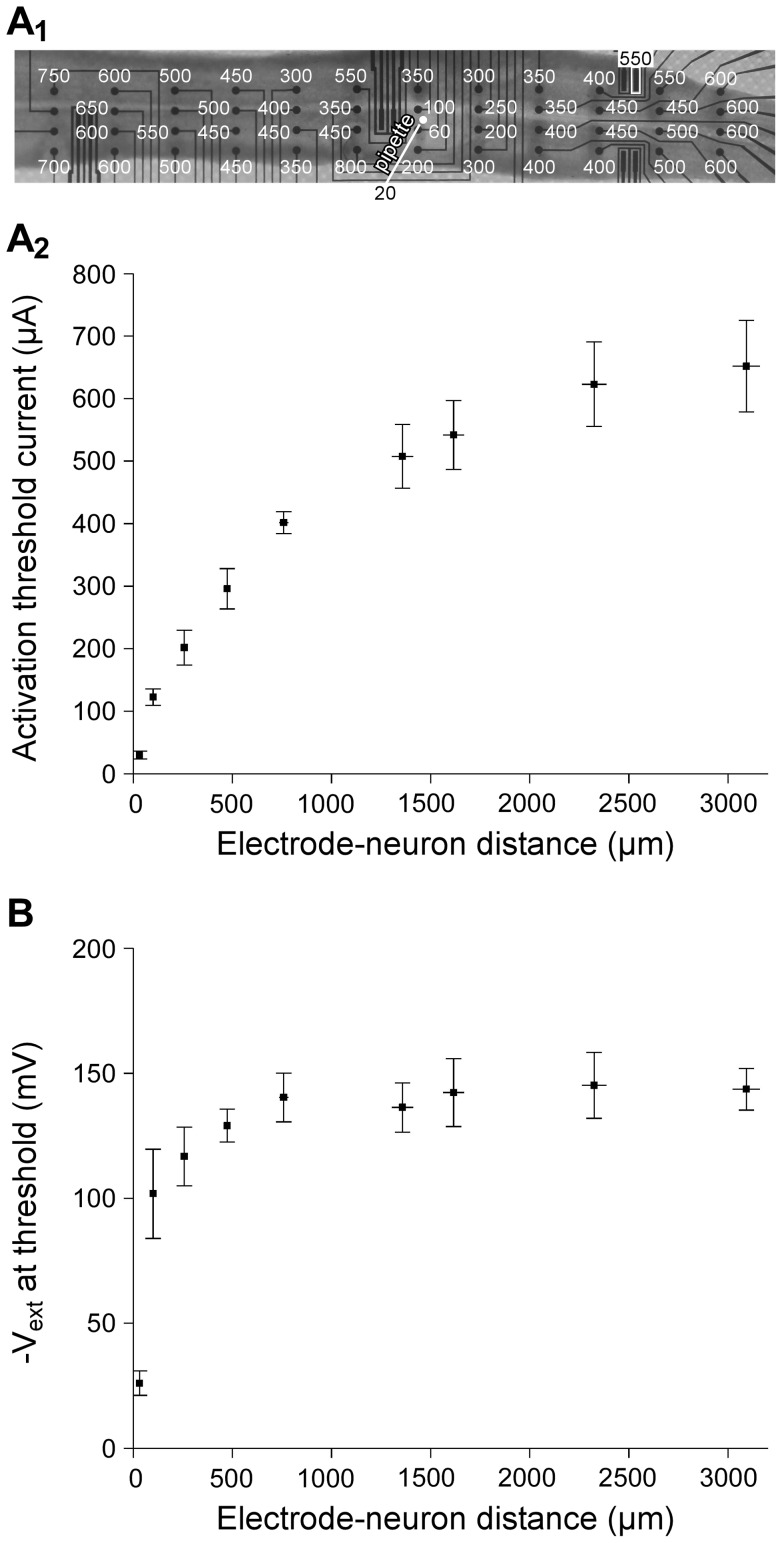
Experimental results. A: Experimental determination of direct activation threshold currents. For each patch-clamped motoneuron, direct activation threshold currents (TCs) were determined by systematically scanning the electrodes of the MEA. A1: Example of thresholds obtained for most electrodes of an array (ranging from 60 to 800 µA) and a stimulating pipette located 20 µm from the soma of the recorded neuron (20 µA). A2: The threshold-distance curve, obtained by pooling the results of 9 experiments, displays a non-quadratic increase of TCs with distance. For instance, on average, TCs reached 200 µA at 250 µm, and 550 µA at 1.6 mm, which is only 2.75 higher. **B: Evolution of the extracellular potential values at threshold with distance.** The amplitude of the extracellular potential field was measured at the end of the cathodic phase of the threshold stimulation. The average potential-distance curve (*n* = 9) shows that this value increases sharply in the first hundred microns and then stagnates at about 140 mV, meaning that action potentials are elicited at the moment when *V_ext_* reaches this value.

Extracellular electrical stimulations were applied to spinal cords of mouse embryos (embryonic age E14.5, *n* = 9) using MicroElectrode Arrays (MEAs). In each experiment, a single motoneuron was patch-clamped in whole-cell configuration, and the threshold current necessary to elicit an action potential was determined for as many electrodes of the array as possible. Figure 3A_1_ gives an example of thresholds obtained for most electrodes of an array and a stimulating pipette located 20 microns from the soma (see Figure 1A) of the recorded neuron. Surprisingly, nearly all electrodes of the array could be used to elicit a spike with currents below 800 µA. Moreover, threshold values were almost independent of the electrode-neuron distance when the electrode was beyond about 1 millimeter from the neuron. This phenomenon was observed across all experiments, as shown in Figure 3A_2_ reporting the average threshold-distance curve obtained from all neurons considered in this study (*n* = 9). On average, we found that the threshold at 2 millimeters (550 µA) was only 2.75 times stronger than that at 250 microns (200 µA). This unexpected result raises a strong issue for achieving focal stimulation of all cells in the vicinity of a stimulating electrode. Indeed, as shown in Figure 1E, thresholds depend linearly on the resting potential of the neuron prior stimulation, and in our case, the slope was found to be about 35 µA/mV. Thus, while a current of 200 µA is required for an electrode to trigger a neuron located at 250 microns with a resting potential of −69 mV, the same stimulus would be sufficient to elicit a spike in a neuron located at 2 millimeters with a resting potential of −59 mV.

Quadratic threshold-distance curves have been introduced based on an initial intuition that the quantity that determines a threshold is the value of the gradient of the extracellular potential at the neuron location. This hypothesis implies that the gradient of the extracellular potential should reach the same value each time the neuron reaches its firing threshold. That is |β∇*V_ext_*|_threshold_ = C, where the constant C does not depend on the stimulating electrode. However, here, we did not find that the gradient of the extracellular potential was constant at threshold. Rather, we found that beyond 750 microns, the value of the extracellular potential itself was nearly constant around *V_ext_* ≈ −140 mV (Figure 3B).

### 2. Modeling results

We next compared these experimental results with modeling predictions. For this purpose, we developed a modeling approach, where we estimated activation thresholds for compartmentalized neurons placed in a potential field calculated using finite element models similar to those already used in the literature [26], [38], [42].

#### a. Discrepancy between experimental and simulated activation thresholds

**Figure 4:**
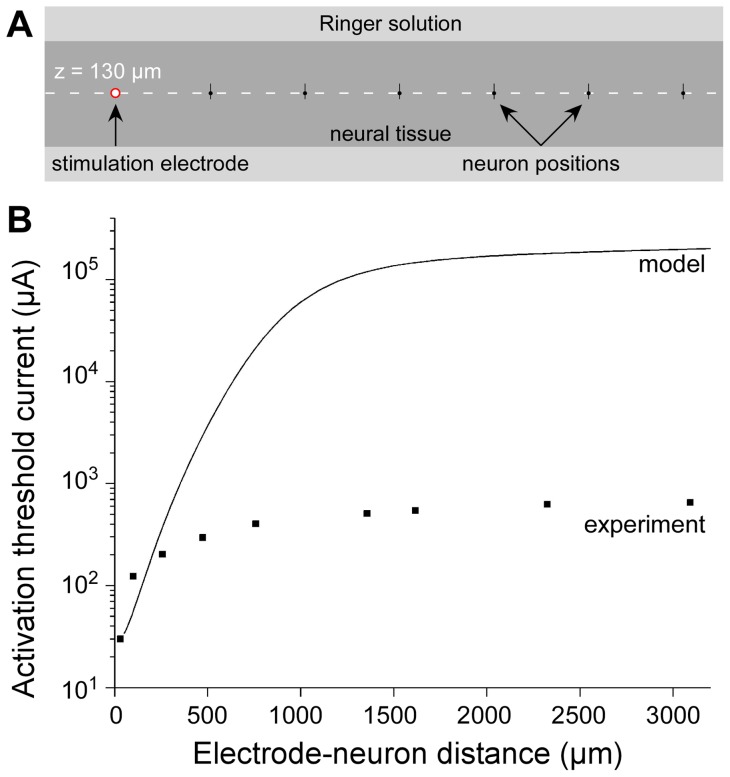
Modeling prediction of activation threshold currents. **A**: Simulated TCs were computed for different positions of Neuron 1 (see Figure 2) along a line passing 50 µm above the tip of the stimulation electrode. **B**: Theoretical TCs (continuous line) were found to be close to experimental TCs (square symbols) for short electrode-neuron distances (typically below 250 µm). For increasing distances, theoretical TCs reached levels several orders of magnitude above experimental ones (note the logarithmic scale).

Threshold currents were computed for different positions of model neuron 1 along a line passing over a stimulation electrode (Figure 4A). We determined TCs for electrode-neuron distances ranging from 50 to 3200 microns (Figure 4B). We found that modeled TCs were close to experimental ones for short distances, in the first hundreds of microns. However, for larger distances (above 1 millimeter), TCs reached and stabilized to values of the order of 10^5^ µA well above experimental thresholds. Thus, simulated thresholds were dramatically over-estimated at these distances (more than 100 times) compared to experimental ones.

#### b. Influence of model parameters on simulated thresholds

**Figure 5:**
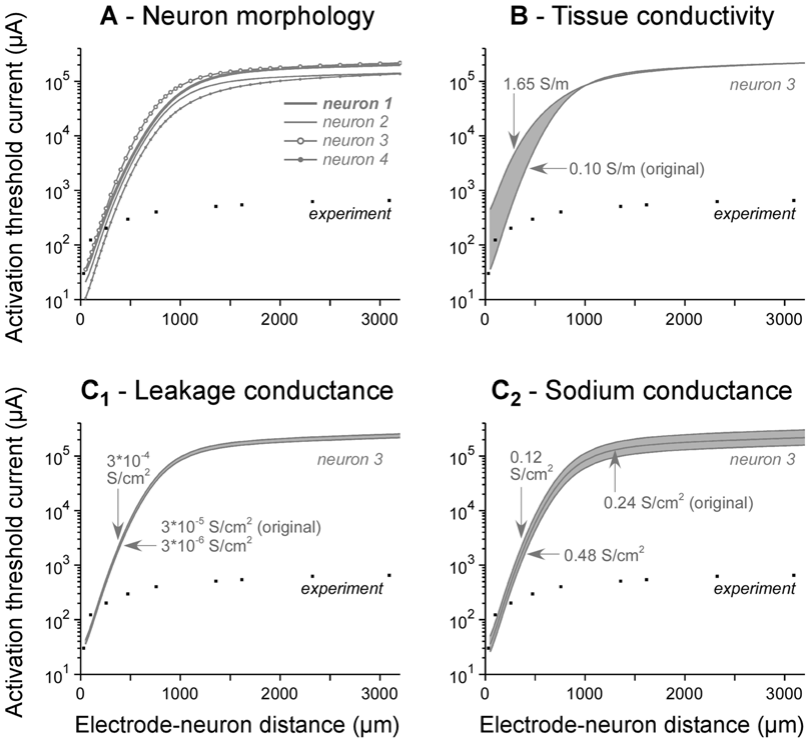
Influence of model parameters on modeling thresholds. A : Different morphologies were considered, from the most complex one (model neuron 1) to the simplest straight fiber (model neuron 4, see Figure 2B). The resulting TC-distance curves were not substantially altered by the choice of the morphology. **B**: Increasing the conductivity of the neural tissue to that of the aCSF (1.65 S/m) increased TCs for short electrode-neuron distances only (below about 1 mm). **C**. Increasing or decreasing the leakage conductance by one order of magnitude only altered the amplitude of the TCs by a factor comprised between −4% and +18.5% (C1). Finally, increasing or decreasing the maximum sodium conductance by a factor of 2 only altered the amplitude of the TCs by a factor comprised between −27.5% and +40% (C2). Overall, modifying any of these model parameters did not change TC values down to levels comparable to experimental ones at large distances (over 1 mm).

We then tested whether some model parameters could be responsible for this strong discrepancy between experiments and modeling, and found that this was not the case (Figure 5). First, we tested the influence of the complexity of the neuron geometry. We replaced the original neuron morphology (model 1) with simpler morphologies made of a reduced number of dendrites (neurons 2, 3 and 4, see Figure 2B_2_). As shown in Figure 5A, this did not affect strongly the simulated TCs. Second, we modified the electrical conductivity *σ_tissue_* of the neural tissue, with values ranging from 0.10 S/m (the original value) to 1.65 S/m (the conductivity of the aCSF solution). Increasing σ*_tissue_* increased TC values for short electrode-neuron distances (Figure 5B), due to the fact that a greater current delivered to the electrode was necessary to achieve similar amplitudes of the electrical potential field. However, TCs were not affected for high distances (above about 1 millimeter). Since neither the neuron morphology nor the tissue conductivity substantially influenced TCs at large distances, we finally tested the influence of the passive and active properties of the neuron. We found that increasing (respectively decreasing) the leakage conductance by one order of magnitude globally scaled the thresholds by a factor comprised between +16% and +18.5% (respectively −3% and −4%) depending on the electrode-neuron distance (Figure 5C_1_, note the vertical shift in logarithmic scale). Similarly, decreasing (respectively increasing) the maximum sodium conductance by a factor 2 globally scaled the thresholds by a factor comprised between +36% and +40% (respectively −26.8% and −27.5%, see Figure 5C_2_). Therefore, modifying the electrical properties of either the neuron or the surrounding medium did not change simulated TCs substantially enough as to explain the strong discrepancy with the experimental TCs.

#### c. Link between the membrane response and the extracellular potential field

To understand why such large currents are necessary to activate modeled neurons at large distances, we determined the link between the profile of the membrane potential *V_m_* and the profile of the extracellular potential *V_ext_* along the neuronal geometry. In a previous study, we showed theoretically that the membrane potential may often be inferred directly from the potential field using the “mirror estimate” [42]:


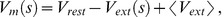
(6)

where *V_rest_* is the resting potential of the neuron and <*V_ext_*> is the spatial average of the extracellular potential field over the neuron morphology, weighted by the local diameters *d*(*j*) of the *N* neuron compartments:


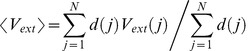
(7)

**Figure 6:**
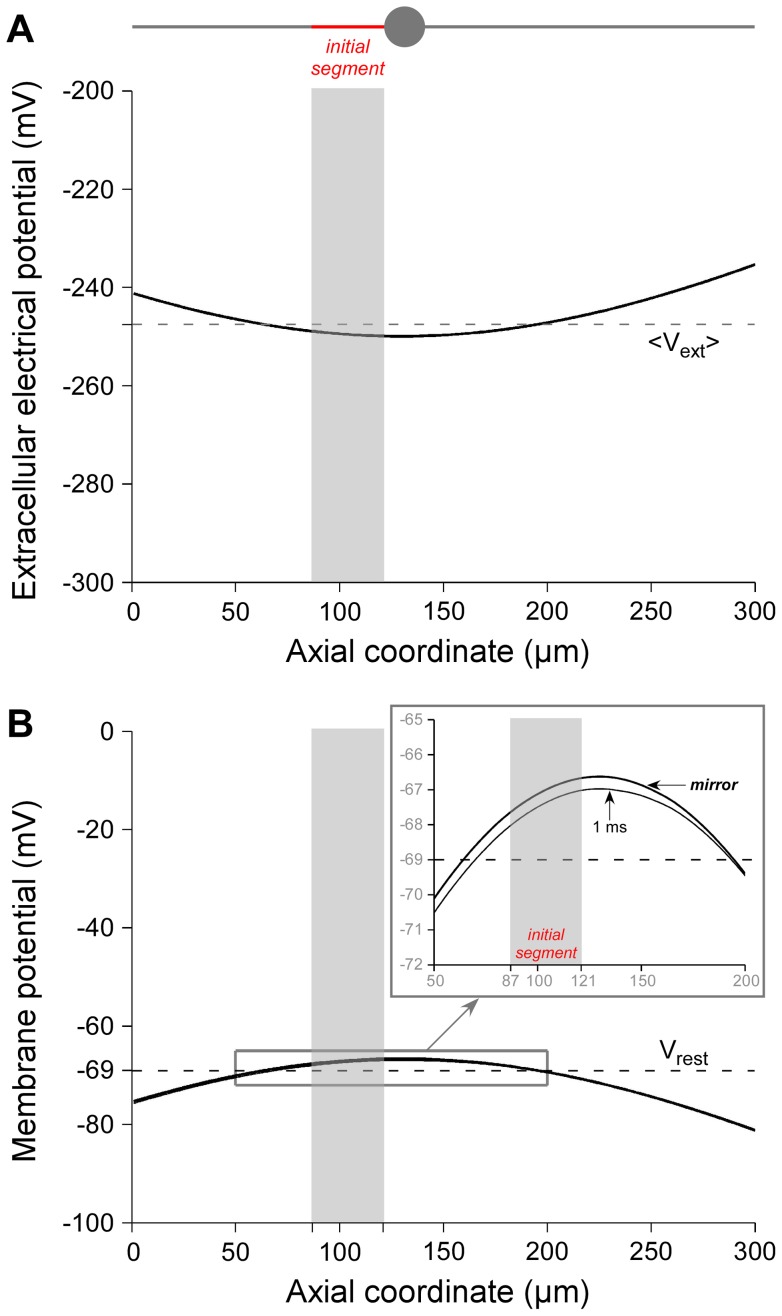
Membrane polarization is well predicted by the *mirror* estimate, and is small at large distances. Longitudinal profiles of extracellular potential *V_ext_* (**A**) and membrane potential *V_m_* (**B**) are plotted for the neuron model 3 (illustrated at the top of the figure), located at 500 µm from the stimulation electrode, for a current of −/+ 442 µA, a level that elicits a spike in experiments. Both profiles are the *mirror* image of each other. The inset in B shows that the membrane potential at the end of the cathodic phase of the stimulus is well predicted by the *mirror* estimate (Eq. 6). It can further be noted that the extracellular potential plotted in panel A displays very small variations around its spatial mean (dashed line). Similarly, the variations of *V_m_* around the resting potential are only of a few mV (B), which is not enough to reach the activation threshold and elicit a spike. At this distance, a current of 6080 µA is actually required to elicit an action potential in the modeled neuron, about 15 times higher than the experimental thresholds.

Here, we verified that the mirror estimate was a good predictor of the membrane polarization in the present case. For sake of simplicity, and because the neuronal geometry did not influence noticeably the TCs at large distances, we considered only Neuron 3, which displays the main elements of the reconstructed motoneuron. Figures 6A shows the profile of *V_ext_* values along the neuron model located at a distance of 500 microns, for a current of −/+442 µA that is large enough to trigger an action potential experimentally (see Figure 3). Figure 6B shows the membrane potential predicted by the mirror estimate and the actual membrane potential at the end of the cathodic phase of the stimulus (1 ms), calculated by solving the full cable equation with the NEURON software. It can be seen that both curves are almost superimposed, assessing the validity of the mirror estimate in this case.

The *mirror estimate* states that the membrane polarization is directly related to how much the potential field fluctuates along the neuron, rather than to the absolute value of the potential itself. At large distances, the potential field around the neuron is nearly constant with only weak spatial fluctuations along the neuron (Figure 6A). Consequently, the membrane potential was also nearly constant. Indeed, as can be seen in Figure 6B, a 442-µA-stimulation induced a fluctuation of the membrane potential of only about 2mV with respect to the resting potential. This explains why reaching the action potential threshold in the neuron model could only be achieved with very high currents (about 6 mA at 500 microns).

#### d. Influence of anisotropic, heterogeneous and dielectric properties of the tissue

Because the mirror estimate holds in the present situation, understanding how the potential field is affected by modifications of the FEM model helps predicting the consequences of these modifications on the neural response. We thus further modified the original FEM model to explore whether anisotropy, heterogeneities or dielectric properties could lead to more ample fluctuations of *V_ext_* along the neuron and thus to smaller TCs, as observed experimentally.

**Figure 7:**
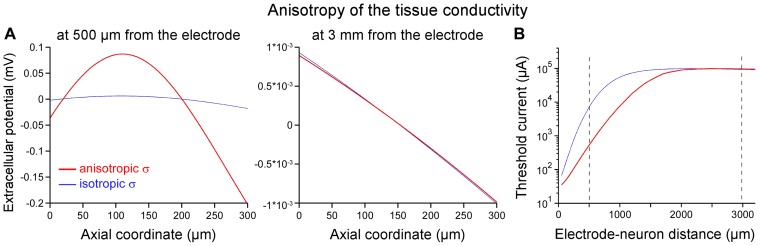
Influence of the anisotropy of the tissue conductivity on the potential field (A) and threshold-distance relationship (B). The originally isotropic tissue model was replaced with an anisotropic volume conductor with a longitudinal (along the *x* direction) conductivity of 0.33 S/m and a transverse (along the *y* and *z* directions) conductivity of 0.083 S/m. **A**: The resulting profiles of the potential field *V_ext_* (centered on its spatial mean) along Neuron 3 (oriented along the *y* direction) are plotted at small (500 µm, left) and large (3 mm, right) distances from the electrode. Large variations of *V_ext_* are observed at small distances, but not at large distances, where the isotropic and anisotropic profiles are almost indistinguishable (compare red and blue lines). **B**: Activation thresholds currents as a function of the electrode-neuron distance for an isotropic (blue) and anisotropic (red) tissue. Consistently with the *V_ext_* profiles shown in A, TCs in the anisotropic case were smaller than in the isotropic case at small distances (541 µA vs. 7280 µA at 500 µm), but almost identical at large distances (96.1 mA vs. 93.4 mA at 3 mm). The 2 vertical dashed lines correspond to the neuron positions at which the *V_ext_* profiles are plotted in A.

First, we replaced the isotropic conductivity of the spinal cord with an anisotropic model of the spinal white matter (see Methods). We found that while the induced fluctuations of *V_ext_* were large at 500 microns (Figure 7A, left), these were very small at 3 millimeters from the electrode (Figure 7A, middle) as with the isotropic model. Accordingly, TCs were reduced for small distances but unchanged at large distances (Figure 7B).

**Figure 8:**
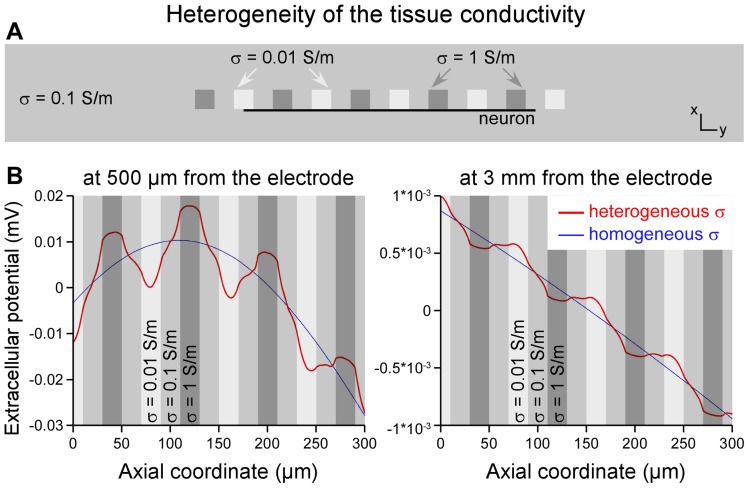
Influence of the heterogeneity of the tissue conductivity on the potential field and threshold currents. **A**: In the originally homogeneous tissue (with *σ* = 0.1 S/m), ten 20-µm-sided cubes were added, in which the conductivity was alternatively set to 0.01 S/m and 1 S/m. The neuron model 3 was positioned at the edge of these cubes to maximize the effect of the heterogeneity. **B**: The extracellular potential field (centered on its spatial mean) is plotted along the neuron model for distances of 500 µm (left) and 3 mm (right) from the stimulation electrode. At 500 µm, the profile of *V_ext_* (in red) displays larger variations than that obtained with the homogeneous model (in blue), with large gradients in the regions of low conductivity (light gray bands) and small gradients in the regions of high conductivity (dark gray bands). These variations result in smaller threshold currents than in the homogeneous model (2.1 mA vs 3.8 mA, respectively). At 3 mm, these field variations are even smaller leading to little decrease of the threshold currents (87.4 mA vs 100.0 mA).

Second, we evaluated the influence of local heterogeneities of the tissue, modeled by embedding small cubic domains with variable conductivity in the spinal cord model (see Methods and Figure 8A). As illustrated in Figure 8B, this induced fluctuations of *V_ext_* along the neuron geometry (red curves) compared to the smooth profile obtained with the homogeneous model (blue curves). However, these variations were small and led to a modest reduction of the threshold currents as compared to the homogeneous model. This reduction was more pronounced at short than at large distances: 2.1 mA vs 3.8 mA at 500 microns from the electrode (Figure 8B, left) and 87.4 mA vs 100.0 mA at 3 millimeters (Figure 8B, right). It should be noted that *V_ext_* variations were maximum along a line passing on the edges of the heterogeneities (Figure 8A) and rapidly vanished away from these edges. Yet, this effect remained far too limited to explain the much smaller threshold currents observed experimentally.

**Figure 9:**
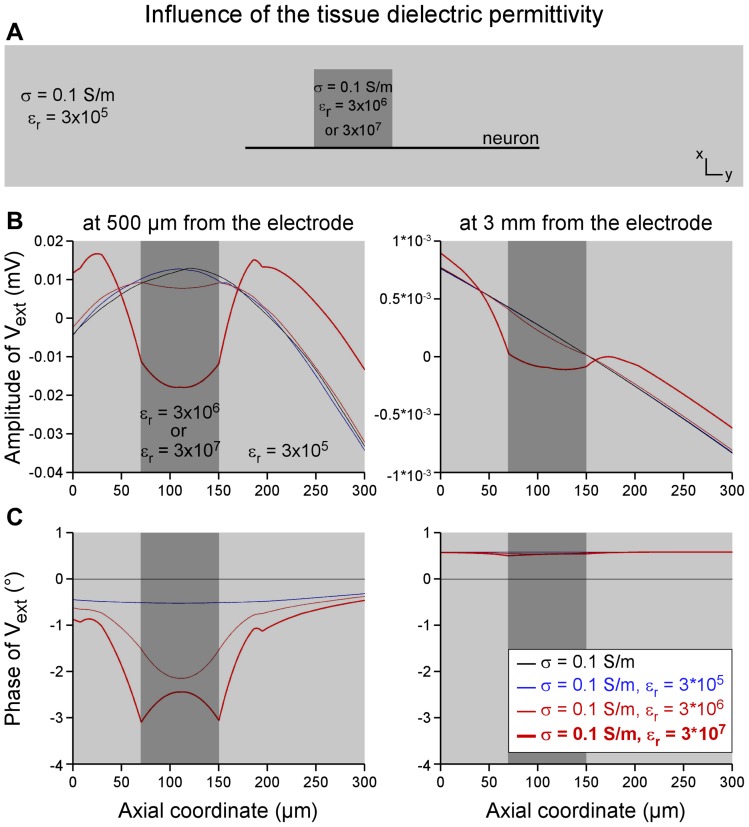
Influence of the dielectric permittivity of the tissue on the potential field. The original purely resistive description of the neural tissue was modified to incorporate its dielectric permittivity ε ( =  ε_0_ × ε_r_), leading to the resolution of the Helmholtz equation instead of the Poisson equation (see Methods, Eq. 5). **A**: The influence of a dielectric anisotropy was also tested by introducing a 80-µm-sided cube with a different relative permittivity value ε_r_ (either 10 or 100 times higher). The neuron model 3 was positioned at the edge of this cube to maximize the effect of the heterogeneity. **B**: The magnitude of the potential field along the neuron (centered on its spatial mean) is plotted for distances of 500 µm (left) and 3 mm (right). Very few differences appear between the homogeneously dielectric (blue) and resistive (black) models. When introducing dielectric anisotropy (red curves), the profile of the field magnitude was modified more importantly for small than for large distances, yet remaining overall within the same range of values as with the homogenous resistive model. **C**: Phase shifts generated by the isotropic dielectric model were uniform along the neuron geometry and very small in amplitude (less than 1°). In the case of the anisotropic dielectric tissue, the phase shift was unchanged at long distance and only slightly modified at short distance.

Third, we tested if the discrepancies between the experimental and modeled TCs could stem from the absence of propagation, capacitive, and inductive effects in the model of neural tissue. To test this hypothesis, we modified the FEM equation to take into account the dielectric permittivity (ε = ε_0_×ε_r_) of the tissue, which led to the resolution of the frequency-dependent Helmholtz equation (see Methods). This equation was initially solved at a frequency of 500 Hz, which is the main component of the spectrum of the biphasic pulse used in this study. As shown in Figure 9B, the resulting potential field had a magnitude very close to that obtained with the resistive model, both at small and large distances (compare the blue and black lines). Moreover, as illustrated in Figure 9C, the phase shift was also extremely limited, with values ranging between −0.5° and +0.5° and remaining almost uniform along the neuron geometry, meaning that the time courses of *V_ext_* were in phase for all compartments (an anti-phase configuration would correspond to a phase shift of 90°). These similarities in magnitude and phase between the resistive and dielectric models were also observed for higher frequencies up to 10500 Hz (not shown).

Finally, we evaluated the effect of possible heterogeneity of the dielectric permittivity, by including a cubic domain in the spinal cord model (see Figure 9A and dark gray bands in Figure 9B), in which ε_r_ was set to either 10 or 100 times its initial value (3×10^5^ at 500 Hz). The first model (ε_r_×10) resulted in electrical potential profiles (thin red curves) close to those obtained with the homogeneous dielectric model, both at short and large distances, and characterized by a small phase shift. Similarly to the homogeneous dielectric model, this was observed at all frequencies. The second model (ε_r_×100) induced larger modifications, essentially on the potential field magnitude and at short distance from the stimulation electrode (Top left, thick red curve). However, at a large distance of 3 millimeters, the amplitude of the potential field along the neuron remained again within its original range, and the phase shift was only very slightly modified.

Overall, taking into account anisotropy, heterogeneities, or dielectric properties of the tissue did not bring noticeable modifications to the potential field that could explain the discrepancies between experimental and modeled TCs.

## Discussion

A major issue in CNS extracellular microstimulation using MEAs is to control the spatial extent of tissue activation. Ideally, each electrode of an array should be considered as an individual stimulation pixel acting exclusively on cells located in its vicinity but not on cells located in the vicinity of neighboring electrodes. However, the spatial influence of an electrode remains largely unknown. Here, we characterized experimentally the spatial extent of monopolar stimulations using MEAs, and compared these results to modeling predictions.

From our experimental results, it appears that, depending on electrode-neuron distance, average currents required to elicit an action potential ranged between 20 µA (at about 20 microns) and 650 µA (at 3 millimeters). These current values fell within the wide range of current values reported in the literature, from a few µA to several mA (for review, see [43]). As a rule of thumb, we found that intensities of about 100–200 µA were required to activate cells within 100–250 microns, which corresponded to an activation spread of the order of 1 µm/µA.

Previous experimental studies have reported that current-distance curves could be well approximated by a quadratic law over distances of a few hundreds of microns [15], [16], [17], [18], [19], [20]. However other work has not confirmed these results. For instance, Abzug and colleagues reported non quadratic but rather linear current-distance curves when spanning distances up to almost 1 millimeter [44]. More recently, fMRI and optical imaging techniques have also been used to determine the spread of activation due to a microstimulation on millimeter-scaled areas [45], [46], [47]. These approaches generally report larger extent of CNS activation than electrophysiological experiments, which can be attributed to the fact that these imaging techniques equally reflect direct and indirect trans-synaptic as well as sub-threshold responses, and not only direct supra-threshold responses. Here, we found that the experimental current-distance relationship for direct activation was not quadratic over distances up to 3 millimeters, and that thresholds actually varied much more slowly than expected.

Although experimental studies provide the only possible way to determine directly the evolution of threshold currents with electrode-neuron distance, such approaches remain difficult to achieve technically, and do not allow an exhaustive screening of different stimulation paradigms or electrode geometries and configurations. For these reasons, computational modeling approaches have been developed to numerically estimate the spatial extent of electrical stimulations [37], [38], [48], [49]. However, to our knowledge, these approaches have not been validated with experimental data directly in a common situation. Here we thus confronted theoretical estimation of activation thresholds with experimental results. We found that simulated TCs corresponded well to experimental TCs for distances smaller than about 250 microns (see Figure 4), with values similar to those found by others (e.g., 200 µA at 150 microns, see [21]). However, an unexpected discrepancy between modeling and experiments was found for distances above about 250 microns, for which simulated thresholds were strongly overestimated (by 2 orders of magnitude at 3 millimeters). We verified that experimental thresholds were not underestimated because of stimulation of distant dendritic or axonal arborization extending in the vicinity of distant stimulating electrodes, as reported in a recent *in vivo* study [47]. Here, this hypothesis could be discarded since the morphology of recorded neurons was found to be much more compact (usually about 300-µm-long, see Figure 2B) than the distances under consideration (several millimeters). Moreover, neurons were chemically isolated from one another using a low-Ca^2+^ aCSF solution, preventing network-induced activation. We also ruled out the possible influence of the neuron morphology and electrical properties (leakage and sodium conductances). Indeed, overestimated simulated thresholds only weakly depended on these model parameter values (Figure 5), a result consistent with other modeling work also reporting robust threshold estimation with respect to model parameters [21].

**Figure 10:**
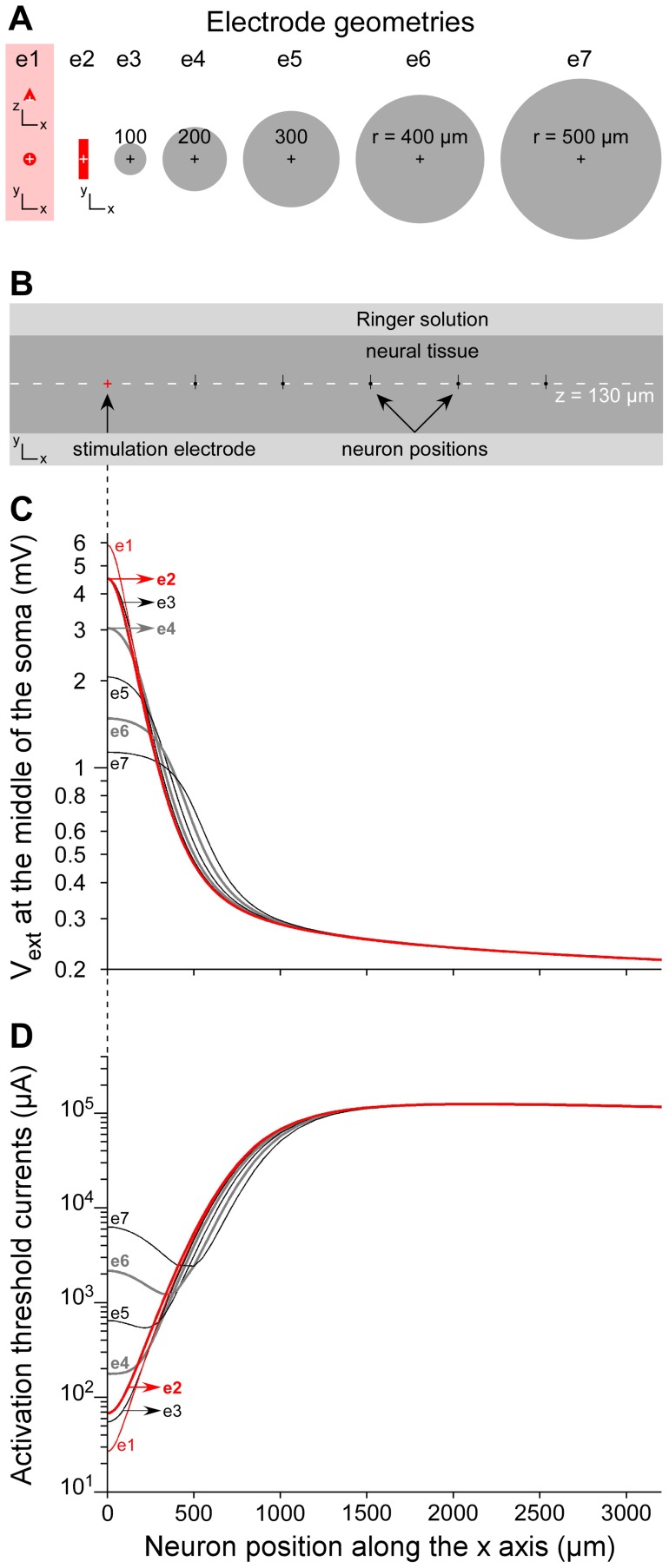
Influence of the electrode shape and size on the potential field and the threshold-distance curves. Seven stimulation electrode geometries were compared (**A**): The original 3D conical electrode used for all simulations of this study (e1, pink area, represented in *x–z* and *x–y* planes), a 2D rectangle electrode (e2, width  = 60 µm, length  = 250 µm) similar to that used in the experimental study, and 5 disk-shaped electrodes of increasing radius (from *r* = 100 µm for electrode e3 to *r* = 500 µm for electrode e7). For each stimulation electrode (centered on *x* = 0), the neuron model 3 was positioned at different locations along the *x*-axis, at *z* = 130 µm (**B**). The extracellular electrical potential *V_ext_*, obtained for a 1-µA-stimulus, was interpolated at the middle of the soma (**C**) and activation threshold currents were computed as a function of the neuron position (**D**). It can be seen that both the potential field and threshold currents were influenced by the electrode shape only for distances smaller than twice the size of the electrode.

In the experimental study, motoneurons were activated with different types of electrodes, including 3D microelectrodes and one larger 2D rectangular electrode. At large distance, the activation threshold currents were similar for these two types of electrodes, which is consistent with the current-controlled mode of stimulation (in Figure 3A_1_, compare the threshold values for the planar rectangular electrode and the 3D electrode on its right on the top row of the array). Accordingly, the modeled thresholds obtained with these two types of electrodes only differed by less than 4% for distances beyond 300 microns, and less than 1% beyond 800 microns. We also further checked that threshold currents obtained with disk-shaped stimulation electrodes of increasing radius reached identical values for distances greater than about twice the electrode's radius (Figure 10). Hence, the electrode size does not influence threshold currents at large distances. This is consistent with a previous modeling study showing that microelectrodes could be well approximated by point sources for distances above a few hundreds of microns [50].

The high values of threshold currents at large distance can be explained by the small variations of the extracellular potential along the neuron geometry. Indeed, we found that the spatial profile of the membrane potential at the end of the cathodic phase was well predicted by the “mirror estimate” (Figure 6), which states that the neuron response relative to the resting potential is equal to the opposite of the extracellular potential centered on its spatial average along the neuron morphology [42]. The relative uniformity of *V_ext_* at large distances, due to the dispersion of the current in all directions of the experimental chamber, thus produced small variations of *V_m_* and hence led to high TCs.

A possible cause of the discrepancy between the experimental and the modeled thresholds could be an oversimplification of the potential field model, based on the Poisson equation and characterized by isotropic, homogeneous and resistive conductivities. We thus further modified this model to embed either anisotropy (Figure 7), heterogeneity (Figure 8) or dielectric properties (Figure 9) in the neural tissue. Some of these features could modify substantially the extracellular potential profiles at small distances (500 microns), and thus (based on the above “mirror” considerations), the neuron polarization and activation threshold currents. However, this was not the case at large distance (3 millimeters), where the model shows important robustness to the different parameters. In particular, the potential field was hardly modified by the dielectric model, which is consistent with a recent modeling study assessing the validity of the quasi-static approximation in the case of extracellular neural stimulation [35]. In this work, the authors indeed found that the propagation and inductive effects were negligible over the considered range of distances (below 1 cm), and that the capacitive effects, although being the most significant, only changed TCs by less than 20% for an homogeneous dielectric tissue. Here, we also tested the influence of local variations of the relative permittivity ε_r_, and did not found substantial changes of the potential field, at least way behind explaining the two orders of magnitude between experimental and modeled TCs at large distances. The most influencing factor was the anisotropy of the spinal cord model, yet insufficient to explain the overestimation of TCs at large distances. Moreover, the embryonic spinal cord being not yet myelinated at the considered embryonic stage (E14.5), such anisotropy is unlikely to take place in our experimental study.

The amplitude of the pulses used in the present study was usually above the safe charge injection limit of Pt microelectrodes (about 25 µA for a 1000-µs pulse and the array that we used, see http://www.qwane.com/Documents/safe_charge_injection_limit.pdf), which could have influenced the neural activity. In this study, every experiment was performed on an acute preparation for only a few hours during which we only delivered a few tens of pulses through each microelectrode. Moreover, although we used stimulations above the safe charge injection limit, we did not observe changes of the threshold currents between the beginning and the end of the experiment (see Methods section 1.d). To further verify the stability of the stimulation, we measured the actual current delivered through the electrode for a given current command, and how this current evolved after repetitive stimulation. We performed such experiments in a Ringer solution, delivering series of –800/+800 µA 1-ms-phase-long biphasic stimuli, and recorded simultaneously the delivered current and the voltage of the electrode. These stimuli corresponded to the highest amplitudes used in our experiments. After 1000 stimuli, we observed no variation of the injected current. Thus, because the extracellular potential field is proportional to the current (and not to the voltage) applied to the electrode [34], stimulations were very stable across the experiments. When delivering these pulses, the potential applied to the stimulating electrode was of the order of 4 V, which was over the safe charge injection limit for platinum (about 1 V). These high voltage values could locally induce water hydrolysis and alter the neural tissue around the stimulation electrode. However, these degradations would mostly influence the response of neurons located close to the electrode. Yet, modeling predictions differed from experimental results only for large electrode-neuron distances but not when the electrode was close to the neuron. Thus, possible overstimulations above the safe charge injection limit could not explain the mismatch between model and experimental results.

In conclusion, two main results are reported in this study. First, we found current-distance curves that were not quadratic over large distances beyond 1 millimeter. Second, we found that simulations predicted threshold currents in accordance with experiments for neuron-electrode distances below 250 microns, but largely overestimated at larger distances. Although the reason for this discrepancy remains to be determined, the present results suggest that activated extents may be underestimated by conventional simulation paradigms. This finding obtained *in vitro* on an embryonic preparation should further be investigated in other types of tissue and also *in vivo* with either MEA microstimulation or more macroscopic deep brain stimulation paradigms.
